# Bilateral Upper Limb Symmetrical Digital Gangrene: A Rare Presentation of Anti-Phospholipid Syndrome

**DOI:** 10.7759/cureus.29516

**Published:** 2022-09-23

**Authors:** Prabhat Rijal, Garima Kaur, Bimalesh Yadav, Rohit Raina, Monika Pathania

**Affiliations:** 1 Internal Medicine, All India Institute of Medical Sciences, Rishikesh, Rishikesh, IND; 2 Psychiatry, All India Institute of Medical Sciences, Rishikesh, Rishikesh, IND

**Keywords:** bilateral, upper limb, symmetrical, anti phospholipid syndrome, digital gangrene

## Abstract

Antiphospholipid syndrome (APS) is an autoimmune disease present most commonly in young women, characterized by the presence of antibodies against various phospholipids and culminating in alteration of the flow of blood, leading to arterial and venous thrombosis. Although it can present with a wide range of manifestations, digital gangrene is one of the important ones. We present a case of a young female with antiphospholipid syndrome who presented with acute onset bilateral upper limb symmetrical digital gangrene with prior history of multiple fetal losses. Acute onset, symmetrical gangrene, limited to the bilateral upper limbs without venous system involvement, that too in association with systemic lupus erythematosus (SLE) which does not usually manifest as such make this case a unique and interesting one.

## Introduction

Antiphospholipid syndrome (APS) is an autoimmune disease associated with the presence of circulating antiphospholipid antibodies, namely, lupus anticoagulant, anticardiolipin antibodies, or antibodies to the protein cofactor b2 glycoprotein I and a variety of clinical manifestations such as vascular thrombosis and pregnancy morbidity [1]. APS can be primary or secondary. The most common etiology of secondary APS is systemic lupus erythematosus (SLE). APS can present with diverse clinical presentations, including symmetrical peripheral gangrene (SPG), which is a rare initial presentation of secondary APS. SPG is frequently omitted in descriptions of clinical aspects of APS and is not mentioned in APS classification criteria. SPG is diagnosed when symmetrical distal ischemia occurs without any large vessel obstruction or vasculitis [2]. We aim to sensitize clinicians to the possibility of secondary APS when we encounter a case of symmetrical peripheral gangrene. Although it is rare, ruling out this entity is crucial and has treatment implications.

## Case presentation

A 29-year-old female housewife from a remote hilly area of Uttarakhand with no known comorbidities and addictions, presented with swelling of the bilateral lower limb for two weeks. The swelling was insidious in onset, which gradually progressed from feet to ankle to legs and finally involving bilateral hands, initially painless, and later associated with a burning sensation in the distal extremities. She developed blackish discoloration of the fingers of the left hand three days after the onset of lower limb swelling, subsequently involving the digits on the right hand within 24 hours. The blackish discoloration was limited to the distal interphalangeal joint in the majority of her fingers and was aggravated by exposure to a cold. However, typical Raynaud’s phenomenon was absent. There was no history of recurrent fever, oral ulcers, genital ulcers, photosensitivity, skin rashes, hematuria, hemoptysis, decreased urine output, frothy urine, jaundice, loss of consciousness, seizures, hemoptysis, cough, chest pain, shortness of breath, history of previous blood transfusion, dizziness, diplopia, sudden deafness, ataxia, or headache.

She had a history of five spontaneous pregnancy losses; the first and second pregnancy losses took place at the sixth month of gestation seven and five years back respectively, the third pregnancy loss occurred at the fourth month of gestation four years back, and the fourth pregnancy loss was at the sixth month of gestation two and a half years back, and the fifth pregnancy loss at the tenth week of gestation four months prior to presentation with above symptoms.

**Figure 1:**
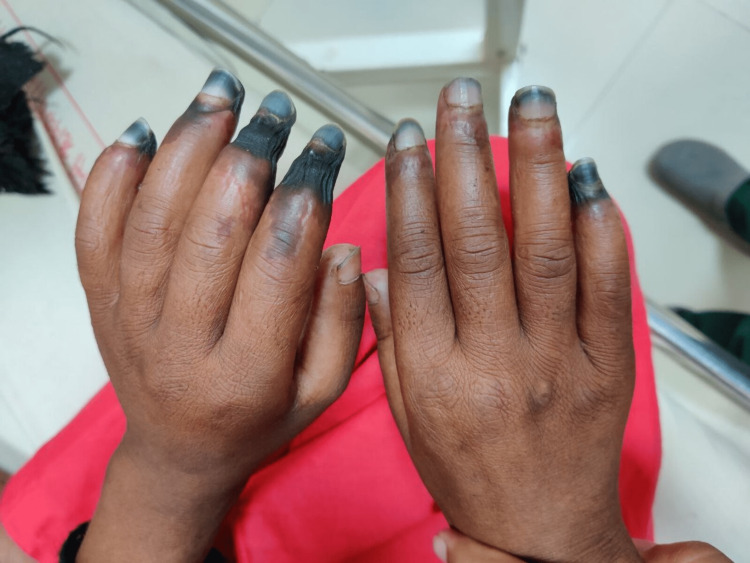
Digital gangrene bilateral hands (dorsal aspect)

**Figure 2:**
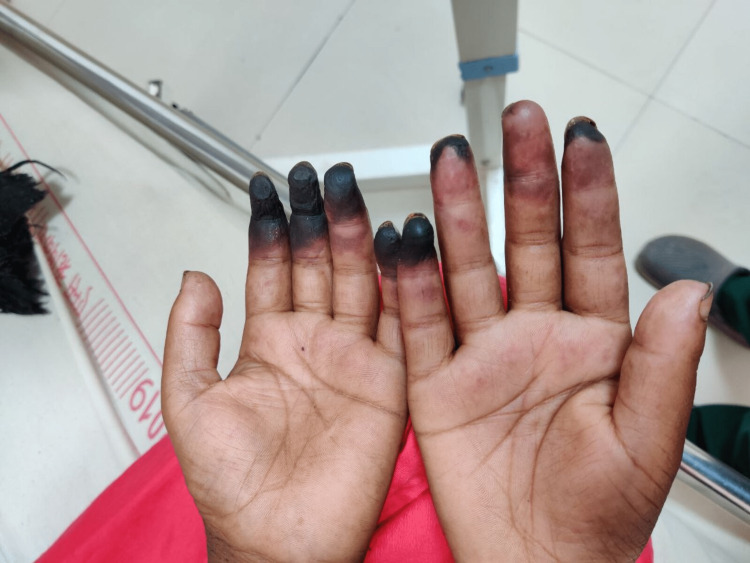
Digital gangrene bilateral hands (palmar aspect)

**Figure 3:**
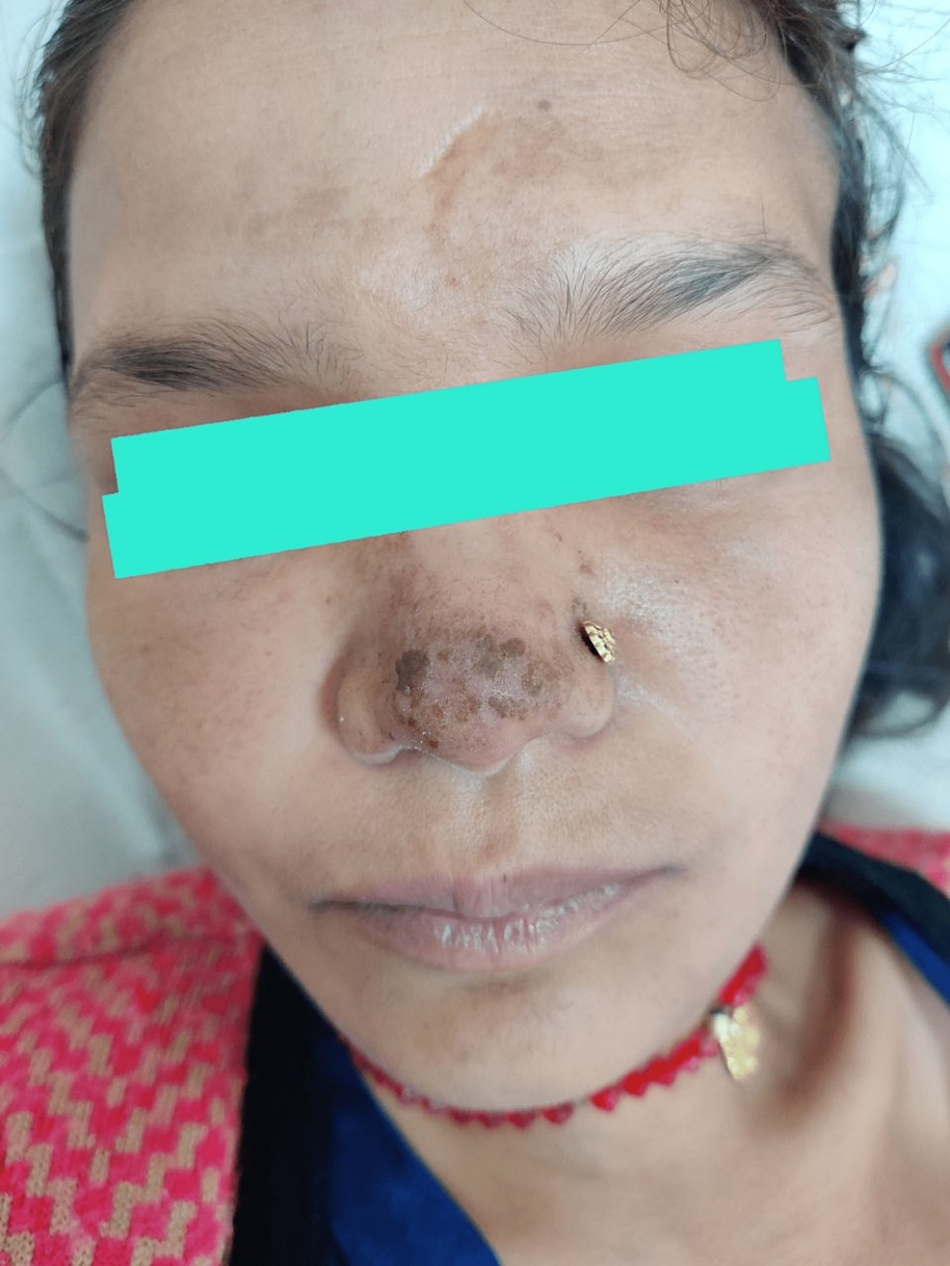
Painless hyperpigmented papular lesion over the tip of the nose

The patient had a regular menstrual cycle with four days of normal blood flow. She took medicine for hypothyroidism six years back, which she discontinued on her own after two years. She presented to the emergency department of our tertiary care center, where her blood pressure (BP) was 90/60 mm Hg at presentation. There was blackish discoloration of all digits of the bilateral upper limb involving the distal half without a line of demarcation (Figures 1, 2). On examination, there was a complete loss of sensation in the blackish area with severe tenderness in the viable area proximal to it. The right radial artery was not palpable, and the left ulnar artery pulsation was also feeble. The lymph nodes were not palpable. However, there was a hyperpigmented papular painless skin lesion over the tip of the nose (Figure 3). With clinical suspicion of APS, a blood sample for antiphospholipid antibody (APLA) profile was sent. Thereafter, the patient was started on oral aspirin tablet and low molecular weight heparin (LMWH). The APLA profile showed prolonged activated partial thromboplastin time (APTT) and diluted Russel viper venom time (DRVVT) (Table 1). Anticoagulation was switched to oral vitamin K antagonist Acitrom (Acenocoumarol) at an initial dose of 2mg once daily, and a target international normalized ratio (INR) of 2.5-3 was achieved during the hospital stay with optimization of dosing of Acitrom (Table 2). The antinuclear antibody (ANA) profile that was sent revealed an ANA titre of 4+ with endpoint dilution of 1:640 and a primary speckled pattern (Table 3) following which an extractable nuclear antigen (ENA) profile was sent. It revealed a strongly positive result for U1 Ribonucleoprotein (U1RNP) (Table 4). After consultation with the rheumatology team, she was started on oral hydroxychloroquine (HCQ) 200mg twice a day, cilostazol 50mg twice a day, and prednisolone 7.5mg once a day. Bilateral upper limbs arteriovenous doppler revealed absent flow in distal right radial and left ulnar artery. CT angiography of the bilateral upper limbs revealed attenuated blood flow in the right radial and left ulnar artery (Table 5). Two-dimensional echocardiography didn’t reveal any significant abnormality. A cardiothoracic vascular surgery (CTVS) opinion was sought, and they advised amputation of the gangrenous digits after the formation of line of demarcation. The patient was discharged from the medicine ward after optimization of medical treatment and attached to CTVS department for amputation of gangrenous digits. In fact, it was a decision that was individualized based on the patient`s circumstances. The majority of the patients with dry gangrene auto amputate, however, there is always a risk of progression to wet gangrene which is more dangerous. As she was from a very remote area with restricted access to a health care centre, it was unlikely for her to get to the hospital in time in case that happens. So the decision of amputation was taken after discussion with the patient and her husband.

**Table 1:** APLA profile APLA: Antiphospholipid Antibodies, DRVV: Diluted Russel Viper Venom, APTT: Activated Partial Thromboplastin Time

APLA components	Result	Normal range
Anti-Cardiolipin IgG Antibody	8.24	<12GPL/ml
Anti-Cardiolipin IgM Antibody	0.98	<12MPL/ml
Anti B2 glycoprotein IgM	3.61	<20RU/ml
Anti B2 glycoprotein IgM	3.69	<20RU/ml
Lupus anticoagulant	absent	absent
DRVV screen time	55.7 seconds	32.8-48 seconds
APTT	64.3 seconds	31.2-41.4 seconds

**Table 2:** General investigations N/L/M/E: Neutrophil/Lymphocyte/Monocyte/Eosinophil, SGOT: Serum Glutamic Oxaloacetic Transaminase, SGPT: Serum Glutamic Pyruvic Transaminase, ALP: Alkaline Phosphatase, GGT: Gamma Glutamyl Transferase, Na: Sodium, K: Potassium, Ca: Calcium, PT/INR: Prothrombin Time/International Normalized Ratio, ESR: Erythrocyte Sedimentation Rate, CRP: C-Reactive Protein, TSH: Thyroid Stimulating Hormone

Investigations	06/04/22	19/04/2022
Hemoglobin (g/dL)	9.4	8.08
Total Leucocyte Counts (per microliter)	10570	7314
Differential Leucocyte Count (N/L/M/E)	71/17/5/1	71/19/8/2
Platelets (10^3^/cumm)	324.8	320.3
Total Bilirubin (mg/dl)	0.11	
Direct Bilirubin (mg/dl)	0.07	
SGOT (U/L)	18	
SGPT (U/L)	10	
ALP (U/L)	101	
GGT (U/L)	26	
Total Protein (gm/dl)	6.6	
Albumin (g/dl)	2.8	
Urea (mg/dl)	24	
Creatinine (mg/dl)	0.48	
Na (mEq/dl)	142	
K (mEq/dl)	4.08	
Ca (mg/dl)	8.9	
Uric Acid (mg/dl)	1.8	
Phosphorus (mg/dl)	4.6	
PT/INR	28.5/2.2	38.7/3.04
Serum C3/C4 (mg/dl)	143/25.1	
ESR (mm/hour)	40	
CRP (quantitative) (mg/dl)	56.8	
TSH (microunit/dl)	2.7	
24-hour urinary protein (mg)	232	
24-hour urinary creatinine (mg)	612	

**Table 3:** ANA profile ANA: Anti-Nuclear Antibody, IFA: Immunofluorescence Assay

Investigations	Result
ANA (IFA)	4+
Primary dilution	1:80
Pattern	Primary speckled pattern
End dilution	1:640

**Table 4:** ENA panel ENA: Extractable Nuclear Antigen, U1RNP: U1 Ribonucleoprotein, SS-A: Sjogren Syndrome-A, SS-B: Sjogren Syndrome B, SCL-70: Topoisomerase I; JO-I: histidyl tRNA synthetase, dsDNA: double-stranded Deoxyribonucleic Acid

Antibodies	Status
Anti-Smith antibodies	Negative
U1RNP antibodies	3+ strong positive
SS-A antibodies	3+ strong positive
RO-52 antibodies	3+ strong positive
SS-B antibodies	Negative
Anti-Histone antibodies	Negative
Anti-Centromere antibodies	Negative
Antibodies to extractable nuclear antigen: SCL-70	Negative
Antibodies to extractable nuclear antigen: JO-1	Negative
Anti-dsDNA	Negative

**Table 5:** Imaging studies HRCT: High-Resolution Computed Tomography, LV: Left Ventricle, CT: Computed Tomography

Imaging Modality	Findings
HRCT Chest	Interstitial Lung Disease-Nonspecific Interstitial Pneumonia pattern
Ultrasonography abdomen and pelvis	No significant abnormality detected
Two-dimesnional echocardiography	Normal LV function, no vegetation or clot
Arteriovenous doppler bilateral upper limbs	No color flow in distal right ulnar artery and distal left radial artery
CT Angiography bilateral upper limbs	Attenuated right ulnar and left radial artery

## Discussion

APS is an autoimmune thrombo-inflammatory disorder involving the circulatory bed in the body. It predominantly affects the deep veins of lower limbs and arterial circulation of the brain, but any tissue can be involved, including vascular beds of the digits leading to digital gangrene [3]. APS is characterized by various forms of dermatological presentations. Among them, distal cutaneous ischemic symptoms due to arteriolar occlusion that culminate in peripheral gangrene are rare. In a case series of 200 consecutive cases of APS, Frances et al. found 49% of APLA patients had dermatological manifestations at the time of presentation, of which livedo reticularis was the most common, found in 25% of patients [4]. In a study including 2,684 lupus patients, those with a long duration of systemic lupus erythematosus (SLE), Raynaud’s phenomenon, and elevated C-reactive protein (CRP) levels were more likely to develop digital gangrene [5]. Our patient also had raised inflammatory markers at the time of presentation. The etiology of digital gangrene in SLE is complex and may involve various factors such as vasculitis, premature atherosclerosis, vasospasm, and thromboembolism. Other contributory factors such as dyslipidemia, disease duration, and elevated CRP can contribute to the pathogenesis of digital gangrene.

APS has been closely associated with lupus digital gangrene; the condition is seen in 3.3%-7.5% of APS cases [6]. There have been only a few reports of SLE presenting initially as digital gangrene. Adelowo et al. have published two cases with digital gangrene as an initial presentation in SLE. The patients were from South Africa [7]. Rosato et al. reported that digital ulcers and gangrene were never present as an initial presentation in SLE [8]; however, multiple other studies have contradicted this. In a clinical image published by Shiba et al. in 2016, they presented a case of a 75-year-old lady with antiphospholipid syndrome who presented with symmetrical digital gangrene involving all four limbs, that developed over a course of two weeks. In that case, the patient had evidence of venous thrombosis in addition to the digital gangrene [9]. Though digital gangrene might not be an uncommon presentation of APS, acute onset with symmetrical distribution, with only upper limb involvement without any venous system involvement, makes our case a unique one. Even if we consider APS to be secondary to SLE, digital gangrene is very less common in SLE compared to other connective tissue disorders like systemic sclerosis.

## Conclusions

Although uncommon, symmetric peripheral gangrene can be the only manifestation of APS, especially when associated with SLE. Clinicians should be able to sensitize themselves to the possibility of secondary APS when they encounter a case of symmetric peripheral gangrene in a young female patient. Timely identification of the disease entity and early initiation of the appropriate anticoagulation therapy is essential to reduce the severity and progression of the disease.
